# Immediate postpartum long acting reversible contraceptive uptake and associated factors at Asella Referral and Teaching Hospital, Ethiopia

**DOI:** 10.1186/s40834-025-00406-y

**Published:** 2025-10-24

**Authors:** Alemtsehay Teshome, Adane Sisay, Amde Eshete, Esayas Regasa, Melkamu Siferih, Daniel Teshome

**Affiliations:** 1 Department of Obstetrics and Gynecology, Salale University, Fitche, Ethiopia; 2 Department of Obstetrics and Gynecology, Arsi University, Asella, Ethiopia; 3 Department of Public Health, Arsi University, Asella, Ethiopia; 4 Department of Obstetrics and Gynecology, Tirunesh Beijing General Hospital, Addis Ababa, Ethiopia; 5 Department of Obstetrics and Gynecology, Debre Markos University, Debre Markos, Ethiopia; 6 Department of Biomedical Science, Salale University, Fitche, Ethiopia

**Keywords:** Long acting reversible contraceptives, Uptake, Post-partal contraceptives, Asella Referral and Teaching Hospital, Ethiopia

## Abstract

**Background:**

Utilizing postpartum long acting reversible contraceptive (LARC) is a key tactic for reducing unwanted pregnancies and increasing birth spacing. The immediate postpartum period is an ideal time to offer LARC methods, as their provision during this period is both safe and effective. Despite these benefits, LARC utilization remains low in Ethiopia. This study aimed to determine the prevalence of immediate postpartum LARC uptake and identify factors associated with its use at Assela Referral and Teaching Hospital (ARTH), Ethiopia.

**Methods:**

A cross-sectional study was conducted through exit interviews with postpartum mothers in the postnatal and post-operative wards of ARTH from August 1 to October 30, 2024. Participants were selected using systematic random sampling from sampling frame. Data were collected via face-to-face interviews using a pretested structured questionnaire administered by trained data collectors with the Kobo Collect tool. The data were then exported to Statistical Package for the Social Sciences (SPSS) version 26 for analysis. Categorical variables were summarized using frequencies and percentages, while means and standard deviations described continuous variables. Descriptive statistics, bivariable, and multivariable binary logistic regression analyses were performed, with statistical significance set at a p-value of less than 0.05.

**Results:**

Out of the targeted sample, 317 respondents completed the study, yielding a response rate of 99.06%. The Magnitude of immediate postpartum LARC uptake was 35.3% (95% CI: 30.3–40.7). The most frequently used postpartum LARC method was Nexplanon (80.4%), followed by Cooper intrauterine devices (IUDs) at 14.3%. Factors such as having ≥ 4 children (AOR = 2.31; 95% CI: 1.01–6.28; *p* = 0.041), cesarean delivery (AOR: 2.14, 95% CI: 1.19–6.72, *P* = 0.032), heard about LARC (AOR = 2.44; 95% CI: 1.43–6.05; *p* = 0.033, previous LARC use (AOR = 5.04; 95% CI: 1.56–9.08; *p* = 0.023), counseling about contraceptives use (AOR = 3.51; 95% CI: 1.45–8.83; *p* = 0.041), and discussions with partners about contraceptives (AOR = 4.03; 95% CI: 2.05–9.41; *p* = 0.024) were significantly associated with LARC utilization during immediate post-partal period.

**Conclusion:**

This study found that approximately one in three women delivering at Asella Referral and Teaching Hospital initiated immediate postpartum long-acting reversible contraceptives (IPPLARC). Factors significantly associated with uptake included having ≥ four children, cesarean delivery, prior awareness and use of LARC, contraceptive counseling, and Male partner involvement in LARC discussions. To improve uptake, health policymakers and hospital administrators should integrate structured contraceptive counselling into routine maternal care, particularly during antenatal care and both cesarean and vaginal deliveries. Health professionals should actively involve male partners and implement targeted awareness campaigns to address misconceptions, especially regarding IUCDs. Context-specific strategies like these can enhance informed decision-making and contribute to higher and more balanced utilization of postpartum LARC.

## Introduction

Family planning (FP) programs are fundamental health services that enable individuals and couples to determine the number and spacing of their children, promoting reproductive autonomy and maternal-child health [1]. Despite the well-documented effectiveness of contraceptive methods in reducing maternal and neonatal morbidity and mortality, a substantial unmet need for modern contraception persists globally [2].

Postpartum contraception is particularly critical in preventing unintended pregnancies and optimizing birth spacing [3]. The World Health Organization (WHO) recommends initiating contraceptive methods within 48 h after delivery to maximize effectiveness and reduce closely spaced pregnancies [4].

Among available options, LARCs, including intrauterine devices (IUDs) and contraceptive implants, are the most effective reversible methods, with failure rates of less than 1% [5, 6].

Implants function primarily through the continuous release of progestin, which suppresses ovulation, thickens cervical mucus, and alters endometrial development [4, 5]. In contrast, IUDs prevent fertilization through distinct mechanisms: levonorgestrel-releasing IUDs inhibit sperm motility and thicken cervical mucus, while copper IUDs induce local endometrial changes and a foreign-body reaction, providing long-term contraception [6, 7].

LARCs are user-independent once inserted, require minimal maintenance, and exhibit higher continuation rates compared with short-acting methods [6].

They are more than 20 times as effective as non-LARC methods in preventing unintended pregnancies and allow rapid return to fertility upon removal [8, 9]. Despite these advantages, global estimates indicate that millions of women experience unintended pregnancies and unsafe abortions annually, contributing to significant morbidity, mortality, and economic burden [10–14].

Globally, there are millions of unintended pregnancies and unsafe abortions each year [10]. Its implications for public health are worrisome [11]. In 2016 alone, WHO reported approximately 282 million unintended pregnancies, 40 million unsafe abortions, and 30 million women facing severe complications, resulting in 5–10 million deaths among women aged 15–49 [12]. It has a high economic cost and result in a 10% reduction in years of disability-adjusted life and production losses [13, 14].

Globally, an estimated 19–20% of women of reproductive age use long-acting reversible contraceptives (IUDs and implants). Utilization shows wide regional variation, with approximately 18% of women in Asia relying on IUDs, and prevalence exceeding 40% in China [6]. According to analysis of the WHO Demographic Health Survey (DHS) of 27 countries, including Ethiopia, many postpartum women have a high unmet demand for FP during the first year after childbirth, but they do not obtain the service [14]. A systematic review found that 28% of pregnancies in Ethiopia were unintended, with the highest rate in the Oromia region at 33.8% [15]. The WHO recommends waiting at least 24 months after childbirth before attempting another pregnancy to help prevent an estimated 30% of maternal deaths and 10% of infant deaths [16].

Nevertheless, DHS data from 21 low- and middle-income countries demonstrated that only 31% of postpartum women used any form of contraception, predominantly short-acting methods (51–96%); in Ethiopia, only 20% of women adopted contraception within two years postpartum, with LARC or permanent methods accounting for merely 12% [17, 18]. The 2019 Mini EDHS further highlighted very low LARC utilization in Ethiopia, with slightly higher rates in urban centers such as Debretabor (3.3%) and West Gojjam (4%), but substantially lower rates in Somali and Afar regions [19].

Despite ongoing efforts by the Ministry of Health, regional health bureaus, and key stakeholders, the uptake of immediate postpartum long-acting reversible contraceptives (LARC) in Ethiopia remains low and highly variable, ranging from 3.3% in Debretabor to 53% at Jimma University Medical Center [19, 20]. Existing studies are limited in scope, often geographically narrow, and sometimes methodologically inconsistent, making it difficult to design effective, targeted interventions at the local level. Importantly, no study has specifically examined the magnitude and factors associated with immediate postpartum LARC uptake in the study area, a setting with unique demographic, cultural, and health system characteristics that may influence utilization.

By focusing on immediate postpartum women at Asella Referral and Teaching Hospital, this study addresses this critical gap, providing context-specific evidence on both the magnitude and factors associated with immediate postpartum LARC use. The findings will generate locally relevant insights to inform evidence-based interventions, strengthen postpartum family planning services, and guide policymakers, health facility administrators, and maternal health programs in designing targeted strategies to improve uptake.

## Methods and materials

This hospital-based cross-sectional study was conducted at Asella Referral and Teaching Hospital (ARTH) from August 1 to October 30, 2024. ARTH, the university hospital of the recently established Arsi University, is located in Asella Town, approximately 175 km southeast of Addis Ababa, and serves as a major referral center for more than 4.5 million population of Arsi Zone and surrounding areas. The zone has an estimated population of 4,326,778, including 805,774 women of reproductive age and 150,133 pregnant and lactating women, with a regional total fertility rate of 5.3 live births per woman. Health services are delivered through 501 health posts, 107 health centers, seven general hospitals, and one referral hospital, with 89% of deliveries attended by skilled birth attendants in 2025, according to the Arsi Zone Health Bureau. Within ARTH, the Department of Obstetrics and Gynecology manages approximately 500–600 deliveries per month, providing 24/7 obstetric and gynecologic care while also serving as a key training center for undergraduate medical students and Obstetrics and Gynecology residents. Family planning services are offered by residents, general practitioners, midwives, and consultant obstetricians and gynecologists.

All eligible and consenting mothers who gave birth at ARTH during the study period were included. However, had undergone tubal ligation or hysterectomy during cesarean delivery, mothers with puerperal sepsis, chorioamnionitis, severe liver disease, and previous breast cancer were excluded. Out of the study participants, three mothers declined to participate, resulting in a response rate of 99.06%.

The sample size was calculated using the Kish formula: n = Z²P (1-P)/W², where *Z* represents the Z-score, *P* the estimated prevalence, and *W* the desired precision. Using a previous LARC prevalence of 25.4% [21], with a 95% confidence interval and 5% margin of error, and adding 10% for possible non-response, the final sample size was 320 participants. To ensure data quality, data collectors received comprehensive training, and data completeness and accuracy were rigorously checked before analysis.

Systematic random sampling was employed to select study participants. The sampling frame consisted of all women who delivered at Asella Teaching and Referral Hospital during the three-month study period (≈ 500 deliveries per month), with an estimated total of 1,500 deliveries. Given the required sample size of 320, the sampling interval (K) was calculated as 1,500 ÷ 320 ≈ 5. A random starting point was selected by lottery from the first five eligible women, and thereafter every 5th woman was approached for inclusion until the desired sample size was attained. Registration of eligible women and application of the sampling procedure were carried out on a daily basis using the delivery register as the sampling frame. Participants were selected systematically using a ***5th*** participants from the sampling frame. If a woman selected through this process did not meet the eligibility criteria, she was excluded, and the next eligible woman was included according to the sampling sequence. Of the women approached, three declined to provide consent, yielding a final sample of 317 participants.

### Variables

**The dependent variable** is **immediate postpartum LARC uptake**, defined as the use of Nexplanon, Jadelle, or IUCD within 48 h after childbirth. It is measured as a binary outcome: **yes** (used) or **no** (not used). **The independent variables** comprised sociodemographic factors (age, income, education, religion, occupation, and residence), obstetric characteristics (parity, mode of delivery, family size, number of live births, and current birth outcome), reproductive intentions (birth planning, number of antenatal visits, and future fertility desires), and contraceptive-related factors (previous family planning use, counseling received, and involvement of male partner in LARC discussion).

### Data collection tools and procedure

To ensure the questionnaire met high standards of validity and relevance, it was developed based on an extensive review of prior studies and literature [3, 5, 6, 10, 12–14, 17, 22–27]. It was initially drafted in English, translated into Afan Oromo (the local language), and then back-translated to English to verify accuracy. A pretest involving 5% of the sample population (16 participants) was conducted at Bokoji General Hospital. As no major modifications were required, data collection was subsequently carried out using the original questionnaire.

Data was collected through face-to-face exit interviews with postpartum and post-operative mothers at Assela Referral and Teaching Hospital (ARTH) between August 1 and October 30, 2024. The process was carried out by five trained BSc nurses stationed in the postnatal and post-op wards.

### Operational definitions

- **Immediate Postpartum Period**: In this study, the immediate postpartum period refers to the first 48 h following childbirth [16].
- **LARC Uptake**: Defined as the use of long-acting reversible contraceptive methods—specifically Nexplanon, Jadelle, or intrauterine contraceptive devices (IUCDs)—by participants during the immediate postpartum period [28].

### Data management and analysis

Data were entered, cleaned, and analyzed using SPSS version 26 (IBM, Armonk, NY, USA).

Descriptive statistics were computed to summarize participants’ socio-demographic, obstetric, and contraceptive-related characteristics. Continuous variables, including age, monthly income, number of children, parity, gravidity, and interbirth interval, were assessed for normality using the Kolmogorov–Smirnov test and visual inspection of histograms. Variables that were approximately normally distributed are presented as mean ± standard deviation (SD), while skewed variables are summarized using median and interquartile range (Q1–Q3). Categorical variables, such as marital status, religion, residence, and contraceptive history, are presented as frequencies and percentages. The outcome variable, was analyzed using binary logistic regression. Bivariate logistic regression was first performed to examine the association between each independent variable and the outcome variables (immediate postpartum LARC uptake (yes/no) with a p-value < 0.25 in the bivariate analysis were included in the multivariable logistic regression model to control for potential confounders. Multicollinearity among independent variables was assessed using the Variance Inflation Factor (VIF). The analysis indicated that parity and number of children were highly correlated, both showing VIF values greater than 10. To address this issue, parity was excluded from the final model, while all other variables demonstrated acceptable VIF values below 6. The adequacy of the final model was assessed using the Hosmer–Lemeshow goodness-of-fit test, which demonstrated good calibration of the model (*p* > 0.05). Adjusted odds ratios (AORs) with 95% confidence intervals were reported, and a p-value < 0.05 was considered statistically significant.

## Results

### Socio-demographic characteristics

A total of 317 pregnant women participated in the study, yielding a response rate of 99.06%. The mean age of participants was 28.9 ± 7.6 years (range: 15–44). Regarding religious affiliation, 48.6% were Muslim and 44.5% were Orthodox Christian. Educational attainment among mothers varied, with college and above graduates accounting for the higher proportion (34.7%), followed by those with secondary education (28.1%). Household income was predominantly below 10,000 Ethiopian birr (ETB) per month (85.5%) **(**See Table 1 for social-demographic characteristics**).**

**Table 1:** Socio-demographic characteristics of mothers who gave birth at ARTH, Asella, Ethiopia, 2024

Variables	Category	Frequency	Percentage
Mothers age	15–24	101	31.9
	25–34	135	42.6
	35–44	81	25.6
Marital status	Married	311	98.1
	Single	4	1.3
	Divorced	2	0.6
Religion	Muslim	154	48.6
	Orthodox	141	44.5
	Protestant	22	6.9
Ethnicity	Oromo	278	87.7
	Amhara	237	11.7
	Gurage	2	0.6
Place of residence	Urban	203	64
	Rural	114	36
Mothers education	College and above	110	34.7
	Secondary school	89	28.1
	Primary school	85	26.8
	Able to read and write	28	8.8
	Unable to read and write	5	1.6
Partners education	Unable to read and write	6	1.9
	Able to read and write	22	7.1
	Primary school	61	19.6
	Secondary school	107	34.4
	College and above	115	37
Mothers occupation	Government employee	135	42.6
	Housewife	78	24.6
	Daily laborer	45	14.2
	Farmer	24	7.6
	Merchant	19	6
	Students	12	3.8
	Others	4	1.3
Partner occupation	Farmer	92	29.6
	Government employee	87	28
	Merchant	86	27.7
	Government employee Driver	19	6.1
	Daily laborer	14	4.5
	Student	13	4.2
Monthly income	< 10,000 ETB	271	85.5
	≥ 10,000 ETB	46	14.5

Others- Broker, driver

### Obstetric profile of the respondents

Regarding parity, 38.2% of the mothers had given birth once, while 22.1% had two births. Whereas, the number of children, the distribution was skewed, with a median of 2.5 children (IQR: 1.6–3.7). The median number of ANC contacts was 3.7 (IQR: 2.8–4.4). Nearly all mothers (99.4%) had attended ANC during their most recent pregnancy. The most common mode of delivery was spontaneous vaginal delivery (SVD), accounting for 57.7% of cases, while cesarean sections comprised 42.3% (Table 2).

**Table 2:** Obstetrics profile of mothers who gave birth at ARTH, Asella, Ethiopia. 2024

Variables	Category	Frequency	Percentage
Parity	One	121	38.2
	Two	70	22.1
	Three	58	18.3
	Four and above	68	21.5
Number of children	One	123	38.8
	Two	75	23.7
	Three	58	18.3
	Four and above	61	19.2
Birth to pregnancy interval	≥ 2 years	122	57
	< 2 years	92	43
Planned pregnancy(current)	Yes	236	74.4
	No	81	25.6
Plan to have more children	Yes	237	74.8
	No	40	12.6
	Undecided	40	12.6
Feature plan of birth	Undecided	157	49.5
	After 2 years	149	47
	Before 2 years	11	3.5
ANC follow up	Yes	315	99.4
	No	2	0.6
Number of ANC contact	Four and above	121	38.4
	Three	85	27
	Two	82	26
	One	12	8.6
Mode of delivery	SVD	183	57.7
	Cesarean section	134	42.3
Birth outcome on discharge	Alive	306	96.5
	Stillbirth	11	3.5

### Awareness and utilization of LARC methods among women who gave birth in ARTH

Most of respondents (63.4%) had heard about LARC methods. Discussion about LARC with family members or others was common, with 60.3% of women engaging in such discussions.

Among those who decided to use family planning, the majority (72.7%) of the respondents made decisions independently, though some reported input from their husbands/parents (50.6%). While 64% of women had never used LARC before, 36% reported previous use. Among all respondents, only 35.3% utilized IPPLARC, while 64.7% did not. The most commonly used LARC method postpartum was Nexplanon (80.4%), followed by cooper IUDs at 14.3%. Family planning counseling was widely accessible during hospital visit, with 88.9% of women reporting they had received counseling. (Table 3).

**Table 3:** Awareness and utilization of LARC methods among women who gave birth in ARTH, Asella, Ethiopia. 2024

Variables	Category	Frequency	Percentage
Heard about LARC	Yes	201	63.4
	No	116	36.6
Involvement of male partners/parent in discussion	Yes	191	60.3
	No	126	39.7
With who do you discussed mostly?	Husband/parents	102	53.4
	Health care provider	65	34.03
	Family member	15	7.8
	Friends	9	4.7
Have you ever used modern contraceptives	Yes	176	55.5
	No	141	44.5
Decision maker to use FP	My self	128	72.7
	Husband/parents	89	50.6
	Health care provider	36	20.8
	Family member	5	2.8
	Religious leader	2	1.1
Have you ever used LARC	Yes	114	36
	No	203	64
Counseled about family planning	Yes	282	88.9
	No	35	11.1
When was the counseling?	During post-partum	127	45
	During labor	92	32.6
	During ANC	63	22.3
Used immediate postpartum LARC	Yes	112	35.3
	No	205	64.7
Type of LARC method used	Nextplanon	90	80.4
	Cooper IUD	16	14.3
	Jadelle	6	5.4

**Fig. 1:**
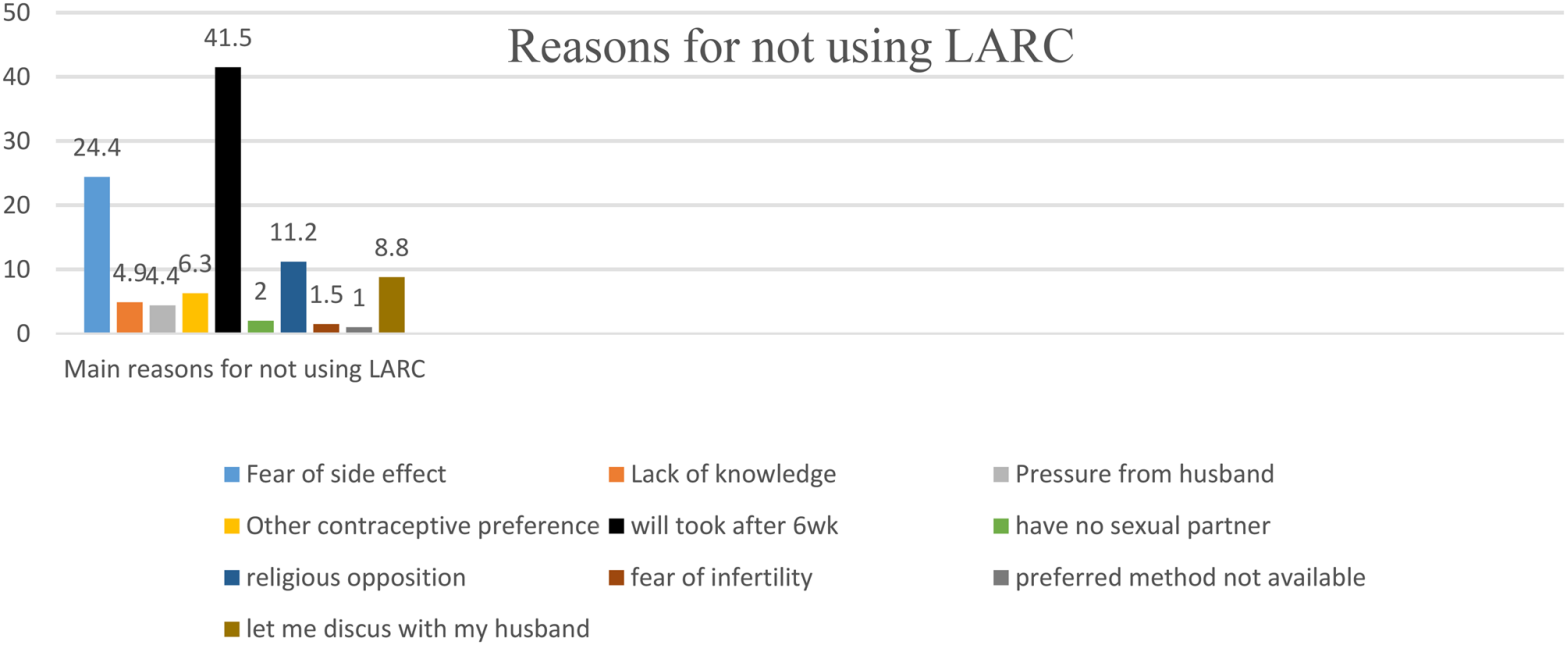
Reason for not using IPPLARC among mothers who gave birth in ARTH, Arsi, Ethiopia, 2024

The main reasons for not taking LARC was preference to take after 6 weeks during immunization (41.5%), followed by fear of side effects (24.4%). (Fig. 1**).**

### Factors associated with utilization of immediate postpartum LARC

The results of the multivariable logistic regression analysis showed that factors such as having ≥ four children, cesarean delivery, heard about LARC, previous LARC use, counseling about contraceptives, and involvement of male partners in discussion about contraceptives were significantly associated LARC utilization during immediate post-partal period.

Women who delivered by cesarean section were more likely to utilize LARC compared to those with vaginal deliveries (AOR = 2.14; 95% CI: 1.19–6.72; *P* = 0.032). Similarly, respondents with four or more children had higher odds of LARC uptake (AOR = 2.31; 95% CI: 1.01–6.28; *P* = 0.041). Awareness and counseling factors were also significant: prior knowledge of LARC (AOR = 2.44; 95% CI: 1.43–6.05; *P* = 0.033), previous LARC use (AOR = 5.04; 95% CI: 1.56–9.08; *P* = 0.023), receipt of contraceptive counseling (AOR = 3.51; 95% CI: 1.45–8.83; *P* = 0.041), and male partner involvement in LARC discussions (AOR = 4.03; 95% CI: 2.05–9.41; *P* = 0.024) were all independently associated with increased LARC utilization. (Table 4).

**Table 4:** Factors associated with utilization of immediate postpartum LARC among women giving birth in ARTH, Ethiopia, 2024 (*n* = 317)

Variables	Category	Utilization of LARC		COR (95% CI)	AOR (95% CI)	P value
		**Yes (%)**	**No (%)**			
Monthly Income	< 10,000	88(27.8%)	183(57.7%)	0.441(0.23, 0.83)	1.64(0.42, 4.84)	0.36
	≥ 10,000	24(7.6%)	22(6.9%)	1	1	
Birth interval	< 2 years	43(20.1%)	49(22.9%)	1	1	
	≥ 2 years	41(19.2%)	81(37.9%)	1.73(0.99, 3.02)	4.98(0.05, 8.14)	0.23
Number of children	One	32(10.1%)	91(28.7%)	1	1	
	Two	28(8.8%)	47(14.8%)	0.59(0.32, 1.09)	0.73(0.051, 3.201)	0.63
	Three	28(8.8%)	30(9.5%)	0.38(0.2, 0.72)	0.67(0.06, 3.39)	0.21
	Four and above	24(7.6%)	37(11.7%)	0.54(0.28, 1.04)	2.31(1.01, 6.28)	0.041*
Mode of delivery	Cesarean section	80(25.2%)	54(17%)	6.99(4.18, 11.69)	2.14(1.19, 6.72)	0.032*
	SVD	32(10.1%)	151(47.6%)	1	1	
Planned to have more children	Yes	70(22.1%)	167(52.7%)	2.38(1.21, 4.71)	0.23(0.04, 3.03)	0.315
	No	22(6.9%)	18(5.7%)	0.82(0.32, 1.97)	2.14(0.59, 8.06)	0.114
	Undecided	20(6.3%)	20(6.3%)	1	1	
Heard about LARC	Yes	97(30.6%)	163(51.4%)	1.66(0.88, 3.16)	2.44(1.43. 6.05)	0.033*
	No	15(4.7%)	42(13.2%)	1	1	
Previous use of LARC	Yes	57(18%)	57(18%)	2.69(1.66, 4.35)	5.04(1.56, 9.08)	0.023*
	No	55(17.4%)	148(46.7%)	1	1	
Counseled about contraceptives	Yes	104(32.8%)	178(56.2%)	1.97(0.86, 4.5)	3.51(1.45, 8.83)	0.041*
	No	8(2.5%)	27(8.5%)	1	1	
Male partner involvement in LARC discussions	Yes	95(30%)	106(33.4%)	5.21(2.91, 9.36)	4.03(2.05, 9.41)	0.024*
	No	17(5.4%)	99(31.2%)	1	1	

## Discussion

This study aimed to evaluate the magnitude of immediate postpartum long-acting reversible contraceptive (LARC) uptake and identify associated factors among women delivering at ARTH, Ethiopia. The findings showed that 35.3% of women adopted LARC methods during the immediate postpartum period (95% CI: 30.3%–40.7%), which can be considered a moderately high uptake when compared with lower rates reported in similar settings, such as [16, 24, 25, 30, 31]. This level of utilization highlights a meaningful opportunity to reduce the unmet need for contraception and to prevent closely spaced pregnancies, both of which are critical for improving maternal and neonatal health outcomes. Several factors were significantly associated with immediate postpartum LARC uptake, including ≥ 4 living children, cesarean delivery, prior exposure to LARC information, previous use of LARC, receipt of postpartum contraceptive counselling, and discussion with a partner about contraception. These findings emphasize the complex interplay of individual, clinical, and relational factors in postpartum contraceptive decision-making and identify key areas for targeted interventions to enhance LARC adoption in similar contexts.

A comparable uptake rate was observed in a study from Durame, Southern Ethiopia [29], where 36.7% of women utilized LARC methods in the immediate postpartum period. The immediate postpartum LARC uptake in our study (35.3%) is comparable to the 30.7% reported in Addis Ababa public hospitals [28], reinforcing the similarity of our findings across comparable settings.The likely reason for this resemblance is that the prevalence of LARC uptake in these places may be influenced by comparable local health education initiatives or similar levels of community awareness regarding LARC, which could be the result of national campaigns.

Conversely, our findings exceed those reported in Hawassa (25.4%) [16], Sidama, southern Ethiopia (21.6%) [25], rural Kenya (20%) [30], East Hararge (18.5%) [21], Mekele, northern Ethiopia (16.4%) [24], West Gojjam (4.02) [31], in Sub-Saharan Africa 12.6% [39], in Nigeria (21.7%) [40] and Uganda (8.5%) [35]. This variation may reflect differences in healthcare provider engagement, with resident doctors and interns actively participating in counseling and timely LARC provision at ARTH. Similarly, Variations in acceptance of contraception may be due to cultural or religious beliefs may partly explain differences between regions.

However, this finding is lower than reports from Jimma, Ethiopia (53%) [23] and Kenya (45%) [32]. The observed discrepancy may be attributed to differences in better community awareness about LARC, study periods, socio-economic characteristics of the study populations, and improvements in health service delivery.

In this study, the most commonly utilized postpartum LARC method was Nexplanon (80.4%), followed by copper IUDs (14.3%). This finding aligns with evidence from Hawassa, Durame where 76.4% and 80% of postpartum women, respectively, opted for implants. However, the use of IUDs in our study (14.3%) was lower compared to Addis Ababa Public Hospitals (24.22%), Hawassa (23.6%) and Durame (20%) [28, 29, 33]. The greater preference for implants, particularly Nexplanon, may be attributed to several factors: its three-year duration of effectiveness, widespread community preference that indirectly influences uptake through peer recommendations, and the tendency of healthcare providers to favor it due to the relative technical simplicity of insertion.

A possible explanation for the lower utilization of IUCD compared with other postpartum LARC methods may be related to both medical and non-medical factors. Clinically, IUCD insertion is contraindicated in the presence of puerperal sepsis, chorioamnionitis, or unresolved postpartum hemorrhage, limiting its eligibility for some women immediately after delivery according to WHO medical eligibility criteria (WHO MEC) [41]. Procedurally, IUCD placement requires speculum examination and insertion through the cervix, which may cause discomfort especially in the early postpartum period and may be perceived as more difficult compared with subdermal implants such as Nexplanon [42, 44]. The device also provides long-term protection (up to 10 years), which may discourage women who prefer shorter-acting methods. Furthermore, common false beliefs and misconceptions such as that IUCD interferes with sexual intercourse, causes infertility or infection, or migrates within the body remain significant barriers in many settings. Provider preference, partner opposition, and community recommendations may also influence uptake, as some providers or peers may be more familiar with or inclined to recommend implants [43].

Nearly two-third (64.7%) of women did not start taking LARC right after giving birth. Preference to start after 6 weeks, fear of side effects and a desire to use other methods were the main reasons for not using postpartum LARCs, accounting for 41.5%, 24.4%, and 6.3% respectively. In a study at Hawassa University Comprehensive Specialized Hospital (22.1% &12.3%) [33], Saint Paul’s Hospital Millennium Medical College (23.5% & 9.8%) [34] and Jimma (25.3% &10.5%) [23], the primary reasons for not using LARC were the desire to begin after six weeks and the selection of alternative approaches.

This study showed that women with four or more children had a 7.6% higher likelihood of utilizing immediate postpartum LARC compared with women who had only one child (AOR = 2.31). This finding is consistent with a study conducted in Farta Woreda, Northwest Ethiopia (8.3%), which also reported higher uptake of IPP LARC among women with increased parity [22]. This may be because mothers with larger families often feel they have reached their desired family size, making them more motivated to adopt long-acting methods for birth limitation or spacing. They also tend to have greater exposure to family planning counseling through repeated health service contacts, better awareness of contraceptive options, and stronger motivation to reduce the economic and social burdens of additional pregnancies, which collectively increase their likelihood of choosing immediate postpartum LARC [22, 35, 44, 45].

Women who had C/S deliveries were also more likely to use LARC (25.2%) than SVD (10.1%), according to the current study. This finding was also reported by studies conducted in Addis Ababa (23.8%) [36] and Kenya (24.1%) [37]. One possible explanation is that women who have had C/S require enough time to heal their scars, as it was strictly advised by medical professionals to stay for at least two years based on the WHO recommendation [38]. As a result, they may be advised to space out their pregnancies or think about their desire to achieve their desired family size. Additionally, a mother delivered by C/S has longer stay in the hospital for at least 48hyrs compared with 6 h for vaginal delivery. Cesarean delivery may facilitate higher immediate postpartum LARC uptake because it creates a clinical opportunity for immediate post-partum LARC utilization and involves multiple provider contacts (preoperative consent, intra-operative and postoperative interactions) that increase counseling opportunities. Empirical studies from diverse settings have observed greater postpartum contraceptive uptake after cesarean delivery and demonstrated that dedicated counselling interventions and routine offering of immediate post-operative LARC increase acceptance [46, 47].

Women who had previously learned about LARC and used LARC techniques were more likely to utilize the service (30.6% and 18%, respectively). This finding is comparable to a study in Farta, Ethiopia, which reported 28.9% and 16.2%, respectively [22], and Kenya (31.8% and 17.3%, respectively [37]showing a similar trend of increased uptake among women with prior knowledge and experience of LARC.

This could be because women who have had prior experience with LACM can receive a plenty of information and advice on the advantages of LARC, its superiority over short-acting treatments, and the costs incurred by healthcare providers. In addition, women who have previously been exposed to LARC may testify about the techniques they employed rather than taking rumours and misconceptions into account [46].

In this study, 32.8% of participants who received postpartum counselling for LARC utilised the service, compared with a lower proportion among those who did not receive counselling. Comparable uptake proportions were also observed in Addis Ababa (30.9%) [27], Hawassa (32.49%) [16], and Eastern Ethiopia (31.9%) [28], highlighting the important role of counselling in improving immediate postpartum LARC use. Counselling may help reduce misconceptions and unfavourable attitudes towards IPPIUCD [43]. One important strategy to address the poor IPPLARC utilisation is to offer counselling to new mothers. Additionally, research emphasises how crucial it is to provide integrated reproductive health services, such as family planning consultation, throughout prenatal, labour, and postnatal visits.

Similarly, women who reported male partner involvement in LARC discussions were more likely to use LARCs (30%). Such findings were reported previously: in Addis Ababa (28.3%) [27], Eastern Ethiopia (27.6%) [28] and (30.2%) Farta Woreda, Northwest Ethiopia [22]. In Ethiopia, where men predominate, a woman’s utilisation of the bulk of reproductive health treatments, including family planning, is contingent upon the approval of men [26]. Additionally, women can choose to space or limit their pregnancies and receive additional support to use maternal health care by talking with their spouses. Couples who receive counselling with their spouses are better able to communicate openly and work together to make family planning decisions. This mutual understanding should lessen misunderstandings and boost support for the use of postpartum LARC in an efficient and timely manner after delivery [27, 28, 43–47].

### Strengths and limitations

A major strength of this study is that it provides timely and context-specific evidence on immediate postpartum LARC utilization in an area where such data were previously scarce. The inclusion of multiple potential determinants, such as counselling, parity, and male partner involvement in LARC discussion, adds depth to the analysis and makes the findings useful for programmatic interventions. This study has some limitations. As a cross-sectional study, it cannot establish causality. Additionally, it was conducted in a single referral hospital and did not include women delivering in health centers, primary, or general hospitals; therefore, the findings may not be generalizable to all postpartum women in the region, including those delivering at home or in lower-level facilities. Finally, reliance on self-reported data may introduce recall or social desirability bias.

### Recommendations

This study underscores the need to strengthen postpartum family planning services, as only 35.3% of women adopted immediate postpartum LARC, with a marked preference for implants over IUCDs. To improve uptake, health policymakers and hospital administrators should integrate structured contraceptive counselling into routine maternal care, particularly during antenatal care and both cesarean and vaginal deliveries. Health professionals should actively involve male partners and implement targeted awareness campaigns to address misconceptions, especially regarding IUCDs. Context-specific strategies like these can enhance informed decision-making and contribute to higher and more balanced utilization of postpartum LARC. To fully understand the complex personal and contextual factors affecting immediate postpartum LARC use, further qualitative research is essential.

## Conclusion

This study found that nearly one-third of women delivering at ARTH initiated long-acting reversible contraception during the immediate postpartum period. Factors such as higher number of children, cesarean delivery, prior knowledge and experience with LARC, counselling on contraceptive options, and male partner involvement in LARC discussions significantly influenced uptake.

## Data Availability

The datasets and materials used and/or analyzed during the current study are available from the corresponding author upon reasonable request.

## Abbreviations

ANC: Antenatal care
ARTH: Asella Referral and Teaching Hospital
CSA: Central Statistical Agency
DHS: Demographic Health survey
EDHS: Ethiopian demographic Health survey
FP: Family planning
IUDs: Intrauterine devices
ICPD: International Conference on Population and Development
IPPFP: Immediate postpartum family planning
IPPLARC: Immediate postpartum long-acting reversible contraception
LARC: Long-acting reversible contraception
LMIC: Low and middle income countries
WHO: World Health Organization
